# Determinants of activity of brown adipose tissue in lymphoma patients

**DOI:** 10.1038/s41598-020-78419-7

**Published:** 2020-12-11

**Authors:** Cornelia Brendle, Norbert Stefan, Eva Grams, Martin Soekler, Christian la Fougère, Christina Pfannenberg

**Affiliations:** 1 Diagnostic and Interventional Neuroradiology, Department of Radiology, Eberhard Karls University, Hoppe-Seyler-Straße 3, 72076 Tübingen, Germany; 2 Endocrinology and Diabetology, Department of Internal Medicine, Eberhard Karls University, Otfried-Mueller-Straße 10, 72076 Tübingen, Germany; 3 Diagnostic and Interventional Radiology, Department of Radiology, Eberhard Karls University, Hoppe-Seyler-Straße 3, 72076 Tübingen, Germany; 4 Oncology, Hematology, Clinical Immunology, Rheumatology and Pulmology, Department of Internal Medicine, Eberhard Karls University, Otfried-Mueller-Straße 10, 72076 Tübingen, Germany; 5 Nuclear Medicine and Clinical Molecular Imaging, Department of Radiology, Eberhard Karls University, Otfried-Mueller-Straße 14, 72076 Tübingen, Germany; 6 Cluster of Excellence iFIT (EXC 2180) “Image Guided and Functionally Instructed Tumor Therapies”, University of Tübingen, Tübingen, Germany; 7 German Cancer Consortium (DKTK), Partner Site, Tübingen, Germany

**Keywords:** Obesity, Radionuclide imaging, Epidemiology

## Abstract

The determinants of brown adipose tissue (BAT) activity are not yet known in detail but might serve as future therapeutic targets against obesity and the metabolic syndrome. We analyzed 235 datasets of lymphoma patients with two PET/CT examinations at different time points retrospectively. We assessed the anthropometric characteristics, features related to the metabolic syndrome, thyroid dysfunction, season of the PET/CT examination, weight change, prior cancer history, lymphoma subgroups, disease activity, and specific lymphoma-related therapies, and evaluated their association with BAT activity. We found BAT activity in 12% of all examinations, and the incidence of BAT activity after initially negative examinations was 10%. In multivariate regression analysis, the prevalence of BAT activity was associated with age, body mass index, sex, the season of the examination, diabetes mellitus, arterial hypertension, and medication on the beta-receptors. New BAT activity arose more often in patients without preceding lymphoma-related therapy. No specific medication was associated with BAT activity. In conclusion, this study confirms the potential connection of BAT with the metabolic syndrome. Preceding lymphoma-related therapy might have an inhibitory effect on the recruitment of BAT.

## Introduction

Activated brown adipose tissue (BAT), a subtype of adipose tissue with high energy consumption, might have a preventive effect against obesity and metabolic disorders^1^. Its physiological role is that of non-shivering thermogenesis by uncoupling ATP synthesis^2^. BAT is the generic term for classical brown adipose tissue, found in newborn mammals, as well as beige fat, which is observed in a small percentage of human adults in the cervical and supraclavicular regions and arises from white adipose tissue^3^. Active BAT was first discovered as an incidental finding in ^18^F-fluorodeoxyglucose positron emission tomography/computer tomography (FDG-PET/CT) examinations for other indications, because of its very high glucose uptake due to extensive metabolic turnover^4–6^. FDG-PET/CT is still the standard method for the identification of BAT. Recent recommendations for a standardized acquisition and evaluation of PET images in this context, the Brown Adipose Tissue Reporting in Imaging Studies (BARCIST) criteria, increase BAT imaging studies’ reproducibility^7^. Several aspects of the physiology of BAT activity in adult humans are unknown, e.g., the precise amount of inactive, but potentially recruitable BAT. Also, the actual impact of BAT activation on weight and metabolic disorders is under discussion, as it constitutes only a small proportion of the whole body tissue^8,9^. The individual physiological circumstances leading to activation of inactive BAT and the formation of new BAT are not completely clear. However, several factors are associated with a higher BAT activity, particularly young age, female sex, low body mass index (BMI), and low blood glucose levels^6,10,11^. In line with its physiological function for thermogenesis, BAT is activated by cold stimuli^6,12,13^. BAT activation works via the uncoupling protein 1, and many potential upstream BAT activators have been proposed based on results from animal studies, computational biology and clinical settings^8,14–16^. In vivo studies in humans identified the beta-receptor agonist mirabegron, melatonin, capsinoids, glucocorticoids, bile acid chenodeoxycholic acid and further dietary components like tea catechins and ephedrine affecting BAT activity^17–25^. Other substances under discussion include thiazolidinedione, resveratrol, thyroid hormones, the intestinal hormone secretin and cytarabine^26–30^. Additionally, active tumor disease itself might be associated with BAT activity^31^.

The rationale behind the search for BAT activating drugs is the hope of detecting potential therapeutic targets to fight obesity and metabolic disorders by increasing the energy consumption and elevating the insulin sensitivity of the treated individuals^1,9,16^. Based on these considerations, the present explorative study aimed to identify the association of BAT activity with patient- and disease-specific parameters in subjects with lymphoma. Lymphoma patients represent a clinical collective with relatively high BAT activity, which possibly facilitates the identification of BAT-activating factors. In this context, we re-evaluated the most familiar potential influencing parameters of BAT activity, as well as lymphoma-related features, in a large patient cohort.

## Results

### Patients

The final dataset contained 235 patients (mean age 50 ± 19 years, 111 females, 124 males). The BMI was higher in males than in females (26.1 ± 4.8 kg/m^2^ versus 24.8 ± 5.4 kg/m^2^, p = 0.005). Age did not differ significantly between both sexes (p = 0.70). Age and BMI showed a weak positive correlation (r = 0.13, p = 0.04). According to the WHO classification, 113 patients suffered from mature B-cell lymphoma, 103 from Hodgkin lymphoma, 11 from other lymphoma or leukemia, and eight from mature T-cell lymphoma (Table 1 shows the detailed histological entities). The mature T-cell lymphoma group contained only males, resulting in a significantly different distribution of the sexes to the groups Hodgkin lymphoma and mature B-cell lymphoma (p < 0.004 and p = 0.01, respectively). The BMI did not differ between the lymphoma subgroups (p = 0.99). Patients with mature B-cell lymphoma (mean age of 60 ± 16 years) were older than patients with Hodgkin lymphoma, other lymphoma, and mature T-cell lymphoma (p < 0.001, p = 0.004 and p = 0.04, respectively).

**Table 1:** Distribution of lymphoma entities in the disease groups.

Entity	N
**Mature B-cell lymphoma**	113
Diffuse large B-cell lymphoma	78
Follicular lymphoma	19
Unspecified B-cell lymphoma	5
Small lymphocytic lymphoma	3
Mantle cell lymphoma	2
High-grade B-cell lymphoma	2
Unclassifiable B-cell lymphoma	1
Mucosa associated lymphoid tissue lymphoma	1
Plasma cell myeloma	1
Primary mediastinal large B-cell lymphoma	1
**Hodgkin lymphoma**	103
**Other lymphoma or leukemia**	11
Lymphoblastic lymphoma of T-cell type	4
Acute myelotic leukemia	3
Unspecified acute B-cell lymphatic leukemia	2
Post-transplant lymphoproliferative disorder	1
Castleman disease	1
**Mature T-cell lymphoma**	8
Unspecified T-cell lymphoma	3
Extranodal natural killer/T-cell lymphoma	1
Peripheral T-cell lymphoma	4

### Prevalence of brown adipose tissue activity

In the whole cohort with 470 PET examinations, BAT activity was present in 55 (12%) examinations, while the remaining 415 (88%) examinations displayed no BAT activity. The median standardized uptake value (SUV) of BAT activity was 2.1, and we categorized accordingly 30 datasets as low BAT activity and 25 as high BAT activity. Table 2 illustrates the relationship between BAT activity and all parameters in detail. Age, BMI, sex, and the season of PET were associated with BAT activity (p < 0.001, p = 0.002, p = 0.01, and p = 0.04, respectively). BAT activity was significantly higher in younger patients, in females, in leaner patients, and in winter compared with summer and autumn (p = 0.02, and p = 0.02, respectively). No patient treated with beta-blockers displayed BAT activity, resulting in a significant difference to patients without beta-receptor therapy (p < 0.001) and receiving beta-receptor agonists (p = 0.03). Diabetes mellitus and hypertension were negatively associated with BAT activity (p = 0.009, and p = 0.003, respectively). All these conditions were independent parameters associated with BAT activity in multivariate regression. In univariate regression, patients with Hodgkin lymphoma had a significantly higher BAT activity than patients with mature B-cell lymphoma (p = 0.004), and chemotherapy with ABVD was associated with a higher prevalence of BAT activity (p = 0.01). Both conditions were no significant factors in multivariate regression. The other assessed features were not associated significantly with BAT activity (details see Table 2). No patient in our cohort received thiazolidinediones.

**Table 2:** Distribution of the study parameters in all PET/CT examinations (n = 470) depending on the BAT activity.

Parameter (patient n)^a^	BAT activity (n or mean ± SD )			Regression (p-value)	
	None	Low	High	Univariate	Multivariate^b^
**Total**	415	30	25	N/A	
**Active BAT depots (range)**	0	2–6	4–6	N/A	
**SUVmean of the active BAT depots**	N/A	1.8 ± 0.2	2.7 ± 0.6	N/A	
**Age (years, n = 469)**	53 ± 18	37 ± 14	28 ± 13	< 0.001*	< 0.001*
**BMI (kg/m**^**2**^**, n = 459)**	25.7 ± 5.3	24.4 ± 5.0	22.6 ± 3.2	0.002*	0.04*
**Sex (females)**	186	21	15	0.01*	0.04*
**Lymphoma group**					
Mature B-cell lymphoma	211	9	7	0.02*	0.13
Hodgkin lymphoma	170	21	15		
Mature T-cell lymphoma	14	0	2		
Other lymphoma	20	0	1		
**Season of PET**					
Spring	112	7	8	0.04*	0.04*
Summer	114	6	3		
Autumn	98	9	2		
Winter	91	8	12		
**Disease activity (n = 422)**	226	12	13	0.58	
**No preceding therapy**	141	10	7	0.83	
**Radiotherapy**	41	3	4	0.62	
**ABVD regimen**	42	8	5	0.01*	0.52
**CHOP/CHLIP regimen**	92	4	5	0.51	
**BEACOPP regimen**	49	6	5	0.23	
**Rituximab**	111	5	4	0.25	
**Cytarabine**	32	1	3	0.48	
**Steroids**	200	13	12	0.88	
**Beta-receptor therapy (n = 462)**					
Inhibition	61	0	0	0.03*	0.02*
No therapy	340	30	24		
Beta-receptor agonist	6	0	1		
**Thyroid dysfunction (n = 458)**					
Hypothyroidism	66	1	4	0.24	
Euthyroidism	315	28	21		
Hyperthyroidism	22	1	0		
**Hyperlipidemia (n = 457)**	43	1	1	0.26	
**Diabetes mellitus (n = 464)**	74	0	0	0.003*	0.004*
**Hypertension (n = 463)**	103	3	1	0.01*	0.03*
**Prior cancer (n = 463)**	37	0	0	0.07	

*Significant, p value of < 0.05, ^a^patient number for evaluation of this parameter if divergent from the total patient number, ^b^only significant parameters of the univariate analyses were included.

*BAT* brown adipose tissue, *SUVmean* mean standardized uptake value, *ABVD regimen* doxorubicin, bleomycin, vinblastine, dacarbazine, *CHOP/CHLIP regimen* cyclophosphamide, doxorubicin, conventional/liposomal vincristine, steroids, *BEACOPP regimen* bleomycin, etoposide, doxorubicin, cyclophosphamide, vincristine, procarbazine and steroids, *N/A* not applicable.

### Incidence of brown adipose tissue activity

A total of 214 patients had one BAT negative PET/CT examination at initial diagnosis or during disease and a subsequent PET/CT examination in the further course, e.g., for therapy evaluation or in suspected relapse and hence were included in this analysis. Incidence of “new” BAT activity in the second examination occurred in 21 cases (10%), of whom 13 patients developed low BAT activity and eight patients high BAT activity. A total of 193 patients remained BAT negative in the follow-up examination. Table 3 illustrates the relationship between the incidence of BAT activity and all parameters in detail. The incidence of BAT activity was associated negatively with age (p < 0.001) and positively with the condition of no preceding lymphoma-related therapy (p = 0.002) in univariate and multivariate regression. New BAT activity occurred more often after therapy with BEACOPP and less common after therapy with rituximab (p = 0.02, and p = 0.02, respectively), but both were not significant in multivariate regression. All other investigated therapies and chronic diseases were not associated with BAT activity in this context (see Table 3). Also, the amount of weight change between the first and second PET/CT examination was not linked with the incidence of BAT activity.

**Table 3:** Distribution of the study parameters in PET/CT examinations of prior BAT negative patients (n = 214) depending on the BAT activity.

Parameter (patient n)^a^	New onset of BAT activity (n or mean ± SD )			Regression (p-value)	
	None	Low	High	Univariate	Multivariate^b^
**Total**	193	13	8	N/A	
**Active BAT depots (range)**	0	3–6	4–6	N/A	
**SUVmean of the active BAT depots**	N/A	1.9 ± 0.2	2.5 ± 0.4	N/A	
**Age (years)**	54 ± 18	40 ± 15	25 ± 10	< 0.001*	< 0.001*
**BMI (kg/m**^**2**^**, n = 209)**	25.9 ± 5.5	25.5 ± 6.6	22.5 ± 3.6	0.22	
**Sex (females)**	84	7	7	0.06	
**Lymphoma group**					
Mature B-cell lymphoma	101	5	0	0.06	
Hodgkin lymphoma	76	8	6		
Mature T-cell lymphoma	6	0	1		
Other lymphoma	10	0	1		
**Season of PET**					
Spring	55	4	2	0.48	
Summer	47	3	2		
Autumn	49	2	0		
Winter	42	4	4		
**Disease activity (n = 189)**	63	3	2	0.73	
**No preceding therapy**	24	6	0	0.002*	0.03*
**Radiotherapy**	27	2	1	0.98	
**ABVD regimen**	21	2	2	0.43	
**CHOP/CHLIP regimen**	64	1	1	0.08	
**BEACOPP regimen**	29	1	4	0.02*	0.86
**Rituximab**	77	2	0	0.02*	0.42
**Cytarabine**	18	1	1	0.93	
**Steroids**	116	5	6	0.20	
**Beta-receptor therapy (n = 209)**					
Inhibition	32	0	0	0.30	
No therapy	152	13	8		
Beta-receptor agonist	4	0	0		
**Thyroid dysfunction (n = 208)**					
Hypothyroidism	34	0	2	0.47	
Euthyroidism	142	12	6		
Hyperthyrodisim	11	1	0		
**Hyperlipidemia (n = 207)**	22	0	0	0.25	
**Diabetes mellitus (n = 211)**	31	0	0	0.13	
**Hypertension (n = 211)**	53	1	0	0.07	
**Prior Cancer (n = 211)**	19	0	0	0.32	
**Weight change (kg, n = 213)**	0.3 ± 7.3	2.5 ± 9.8	− 0.9 ± 13.9	0.65	

*Significant, p value of < 0.05, ^a^patient number for evaluation of this parameter if divergent from the total patient number, ^b^only significant parameters of the univariate analyses were included.

*BAT* brown adipose tissue, *SUVmean* mean standardized uptake value, *ABVD* *regimen* doxorubicin, bleomycin, vinblastine, dacarbazine, *CHOP/CHLIP regimen* cyclophosphamide, doxorubicin, conventional/liposomal vincristine, steroids, *BEACOPP regimen* bleomycin, etoposide, doxorubicin, cyclophosphamide, vincristine, procarbazine and steroids, *N/A* not applicable.

## Discussion

In this study, we evaluated the prevalence and incidence of BAT activity in lymphoma patients. We assessed various potential determinants of BAT activity to gain further insights into the modulation of BAT as a potential therapeutic target against obesity. While the BAT prevalence might relate primarily to strong known impact factors and chronic conditions, the investigation of new incidence of BAT activity might reveal the potential impact of temporary factors. Our patient cohort showed a similar distribution of age, sex, and BMI to prior unselected studies on BAT activity^6,10,32^. Therefore, our cohort might compose a representative sample of a lymphoma population. The prevalence of BAT activity was 12% in our study, and the incidence of BAT activity in initially BAT negative patients was 10%. Both rates are higher than reported ratios of about 4–5% BAT activity in mixed populations^6,10,32^. In agreement, other studies with pediatric and adult lymphoma patients observed a comparatively high prevalence of BAT activity^6,30,33,34^.

As expected, BAT activity was associated with its well-known determinants age, sex, BMI, and season^6,10^. Additionally, our results strengthen the potential role of BAT in the metabolic syndrome by showing a negative association with diabetes mellitus and arterial hypertension^6,10,35^. We found an analogy between BAT and beta-receptors' activity, confirming prior findings where treatment with beta-blockers and beta-receptor agonists affected BAT^6,17^. On the other hand, BAT activity was not independently associated with other factors under discussion, like hyperlipidemia, thyroid dysfunction, prior cancer disease, and the specific lymphoma type^1,16,28,33,35^.

We found no association of BAT and lymphoma’s metabolic activity in our cohort. Former studies reported variable results on the role of cancer vitality in the regulation of BAT activity^31,33,36^. Also, BAT activity was not linked with the extent of weight change during the disease, in contrast to prior reports on cancer cachexia^35^. Thus, other disease accompanying conditions, particularly the application of a specific therapy, might be relevant. The effect of preceding chemotherapy on BAT activity is controversial^10,30,37^. The present results plead for an inhibitory role of chemotherapy or radiotherapy on BAT since the BAT recruitment was higher in patients without preceding lymphoma-related therapy. Steroids have been reported to stimulate BAT activity in the short-term but inhibit its function during long-term use. The real impact of steroids on energy metabolism via regulation of BAT activity is unclear, and we did not find an association with BAT activity in the present study^21–23,30,38^. Also, cytarabine application did not affect BAT activity, in contrast to a previous study with a comparable patient cohort and although it shares the signaling pathway with BAT^30^. The impact of cytarabine on BAT activity might be minor and prone to interference by other influencing factors. Furthermore, the investigated patient numbers are low so far, and the potential role of this medication on BAT activity can be answered only after larger studies. The ABVD and BEACOPP regimen contain doxorubicin and vincristine, potential BAT recruiting agents^30,39^. Although we saw a tendency towards altered BAT activity by applying these chemotherapies, we could not independently prove an association from confounding factors. Further studies are needed on these medications as well.

Our study has some limitations due to its retrospective design and the limited numbers of patients in specific subgroups. Therefore, we could not consider specific lymphoma entities, all single drugs, and all potential confounders in our analyses. Furthermore, the patients did not undergo a cooling protocol or a preparatory high fat diet before the PET/CT examinations, which both increase BAT activity. In the assessment of BAT activity by FDG PET, the differentiation between active BAT and other conditions with metabolic activity, e.g., lymph nodes, can be challenging due to the unspecific nature of FDG uptake. Our approach, according to the BARCIST criteria, aimed to minimize this bias by a fixed lower cut-off value and a specified HU range in CT for defining BAT as well as drawing the VOI in a sufficient distance to visible lymph nodes^7^. Continuous quantification of BAT activity is not possible using a fixed cut-off value. Therefore, we differentiated two BAT activity levels based on the median SUV of patients with BAT activity, but they are still artificial. Finally, including only two PET/CT examinations per patient might introduce selection bias, but it results in a more balanced dataset than including different examination numbers per patient.

In conclusion, BAT activity in lymphoma patients is related to the well-known anthropometric factors, conditions of the metabolic syndrome, and the beta-receptors' activity. While we did not find a connection between BAT activity and a specific medication of lymphoma, preceding lymphoma-related therapy might have an inhibitory effect on BAT recruitment.

## Methods

### Study design and patients

We retrospectively evaluated all lymphoma patients who underwent two or more ^18^F-FDG-PET/CT examinations between January 2012 and December 2018 in the PET/CT center of our university hospital. In patients with more than two PET/CT examinations, we included either the last PET/CT without BAT activity and the first examination with BAT activity, or—if not applicable—the first two examinations in the disease course. We assessed age, sex, and BMI and categorized the lymphoma entities according to the WHO classification in the four groups mature B cell-lymphoma, Hodgkin lymphoma, mature T cell-lymphoma, and other lymphoma^40^. We identified the lymphoma disease as active if vital manifestations with FDG uptake above the mean liver uptake were present. Cases with unclear faint FDG uptake in morphologic residual caused by minimal residual activity or reactive changes were not considered. We assessed the season at the PET/CT examination and calculated the weight change between the two examinations. We noted, which therapy regimen was applied before the respective PET examination since the beginning of the disease or a former PET examination. We considered specific medications if more than thirty individuals received them. This criterion applied to the chemotherapy regimens ABVD (doxorubicin, bleomycin, vinblastine, dacarbazine), CHOP/CHLIP (cyclophosphamide, doxorubicin, conventional/liposomal vincristine, steroids), and BEACOPP (bleomycin, etoposide, doxorubicin, cyclophosphamide, vincristine, procarbazine, steroids), as well as the separate drugs rituximab, cytarabine and steroids. We waived infrequently used drugs due to the limited statistical power. We searched the patients' history concerning the following further factors potentially linked with BAT activity: prior cancer disease, arterial hypertension, hyperlipidemia (known disease or treatment with statins), medication on the beta-adrenergic receptors (separate categories for beta-blockers, or beta-receptor agonists), and thiazolidinediones. We noted thyroid dysfunctions (differentiation in hypo- or hyperthyroidism) and diabetes mellitus if they were reported as diseases, a specific medication was applied, or the corresponding blood values were abnormal at the PET/CT examination (normal range of thyroid-stimulating hormone 0.2–4.2 mU/l, and fasting blood glucose < 7.0 mmol/l). The study was approved by the ethics committee of the university hospital of Tuebingen. All patients gave their written informed consent for the scientific use of their data. The study was performed in accordance with the Declaration of Helsinki 2013 and based on the principles of the International Conference on Harmonization: Good Clinical Practice guidelines.

### PET/CT examinations and image analysis

**Figure 1:**
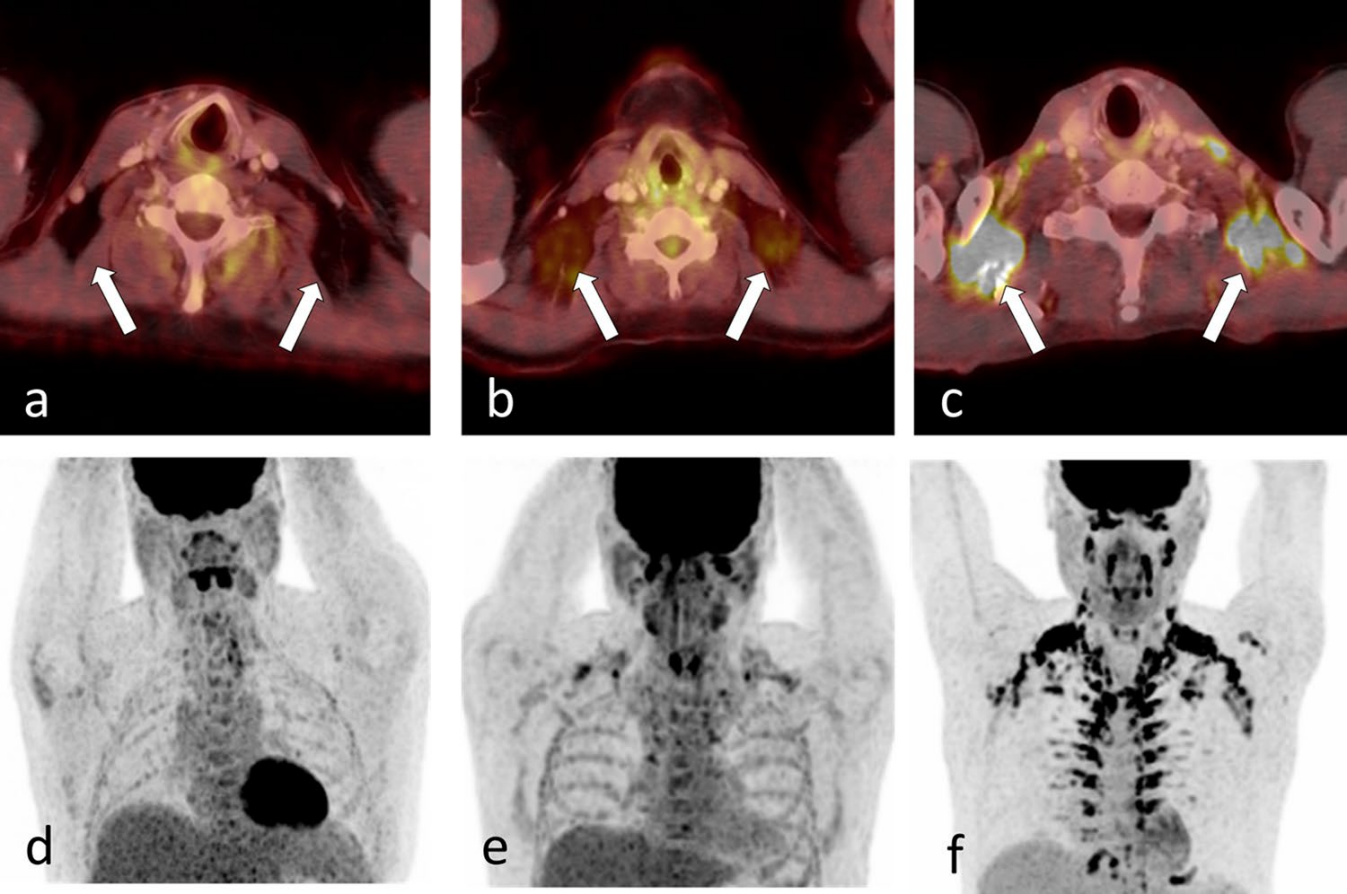
Categories of BAT activity. Axial fused PET/CT images (**a**–**c**) and maximum intensity projection of PET (**d**–**f**) of patient examples with no activity of brown adipose tissue (BAT, **a**,**d**), low BAT activity (**b**,**e**) and high BAT activity (**c**,**f**, see arrows).

All patients underwent the ^18^F-FDG-PET/CT examinations on a Biograph mCT (Siemens Healthcare, Knoxville, USA) under standardized conditions with a constant temperature of 22 °C. The PET/CT protocol contains a multidetector CT with a peak voltage of 120 kVp, a tube current of 250 mAs, and a table feed of 31 mm. All patients had fasted at least 6 h before the examination. The patients rested during the uptake time for 60 min in an air-conditioned waiting room with a constant temperature of 22 °C after the injection of 322 ± 4 MBq ^18^F-FDG. PET data were acquired from the skull base to the thighs (acquisition time 2 min/bed, 6–8 bed positions) and reconstructed with a 3D ordered-subset expectation maximization (OSEM) algorithm (2 iterations, 21 subsets, Gaussian filter 2 mm). CT data were reconstructed in the axial direction (slice thickness 3 mm, increment 2.5 mm) and used for image analysis and attenuation correction. The commercial software SyngoVIA (Siemens Healthcare, Knoxville, USA) served as the study's viewing platform. We used a standardized uptake value (SUV) normalized on lean body mass for PET quantification. We reviewed the fused PET/CT images of all patients for BAT activity in the right and left nuchal, supraclavicular, and mediastinal regions according to the BARCIST criteria. We quantified BAT activity using isocontour volumes of interest (VOI) with a lower cut-off SUV of 1.2 if the region's density corresponded to fat in CT (Hounsfield Units − 10 to − 190)^7^. FDG positive lymph nodes near the areas of BAT assessment are a potential finding in lymphoma patients. We took special care to avoid spillover bias by adhering to a sufficient distance between visible lymph nodes and each VOI. We did not quantify regions with an SUV below the cut-off value, as these do not represent BAT activity according to BARCIST. In each patient, we noted the number of measurable VOI (active BAT depots) and averaged the mean SUV (SUVmean) of all active BAT depots. Subsequently, we classified the extent of BAT activity in three categories to reflect the continuous nature of BAT activity: high BAT activity (≥ 4 active BAT depots and SUVmean above the median SUVmean of all patients with BAT activity), low BAT activity (measurable BAT activity, but below the criteria for high BAT activity), no BAT activity (examples see Fig. 1). EG and CB read all PET/CT images in consensus (1 and 10 years of experience in hybrid imaging analysis).

### Statistical analysis

Shapiro–Wilk test denied the normal distribution of the data. The variables were categorical with a nominal or ordinal scale, except for the continuous variables age, BMI, and weight change. We tested the association between continuous and categorical variables with univariate logistic regression and Wilcoxon test, and the association between categorical variables with the Chi-Square test. We used Spearman correlation to test the relation of continuous variables. We identified independent parameters associated with BAT activity by multivariate regression with all significant parameters. We set the significance level at a p value ≤ 0.05 and used JMP 13.2 as statistical software (SAS, Cary, USA).

## Data Availability

The datasets analyzed during the current study are available from the corresponding author on request.
